# Neuropathophysiology of Lysosomal Storage Diseases: Synaptic Dysfunction as a Starting Point for Disease Progression

**DOI:** 10.3390/jcm9030616

**Published:** 2020-02-25

**Authors:** Camila Pará, Poulomee Bose, Alexey V. Pshezhetsky

**Affiliations:** 1 CHU Sainte-Justine Research Center, University of Montreal, Montreal, QC H3T 1C5, Canada; camila.debrittoparadearagao@mail.mcgill.ca (C.P.); poulomeebose@gmail.com (P.B.); 2 Department of Anatomy and Cell Biology, McGill University, Montreal, QC H3A 0C7, Canada; 3 Department of Pediatrics, University of Montreal, Montreal, QC H3T 1C5, Canada

**Keywords:** Lysosomal storage diseases, synaptic dysfunction, synaptic spines, mucopolysaccharidosis, Batten disease, NCL, Niemann-Pick type C, gangliosidosis, Krabbe disease

## Abstract

About two thirds of the patients affected with lysosomal storage diseases (LSD) experience neurological manifestations, such as developmental delay, seizures, or psychiatric problems. In order to develop efficient therapies, it is crucial to understand the neuropathophysiology underlying these symptoms. How exactly lysosomal storage affects biogenesis and function of neurons is still under investigation however recent research highlights a substantial role played by synaptic defects, such as alterations in synaptic spines, synaptic proteins, postsynaptic densities, and synaptic vesicles that might lead to functional impairments in synaptic transmission and neurodegeneration, finally culminating in massive neuronal death and manifestation of cognitive symptoms. Unveiling how the synaptic components are affected in neurological LSD will thus enable a better understanding of the complexity of disease progression as well as identify crucial targets of therapeutic relevance and optimal time windows for targeted intervention.

## 1. Introduction

Lysosomal storage diseases (LSD) are progressive inherited disorders of metabolism associated with accumulation of biological macromolecules or products of their catabolism inside the organelles of the endosomal-autophagic-lysosomal system, and caused by a genetic deficiency of lysosomal enzymes and proteins involved in the functioning or biogenesis of lysosomes. The concept of a biochemical mechanism of LSD has been first provided by H. G. Hers [1] who discovered that deficiency of acid α-glucosidase causes accumulation of glycogen in several tissues of patients affected with Pompe disease (glycogen storage disease type II), a severe hereditary disease manifesting with cardiomyopathy and muscle weakness [2]. 

Symptoms frequently associated with LSD include dysmorphic features, musculoskeletal abnormalities, organomegaly, seizures, hypotonia, ataxia, progressive cognitive and motor retardation, and hydrops fetalis in severe cases [3]. However, the clinical phenotype, the age of onset and the spectrum of presented symptoms show remarkable variability depending on the type of stored material, nature of the cell/tissue where the storage occurs, and the extent of catabolic dysfunction caused by specific mutations [4]. More than 50 LSD have been described in humans, the majority of which are caused by mutations affecting lysosomal hydrolases. However, defects in the export of catabolites or vesicular trafficking may also lead to the lysosomal storage phenotype [5]. Besides, LSD can be also caused by defects in non-enzymatic lysosomal proteins, involved in protection/activation of lysosomal enzymes, their post-translational processing, or trafficking to the lysosome as well as lysosomal membrane channels and transporters necessary to maintain the function of the lysosome [6]. For instance, one of the causative genes for neuronal ceroid lipofuscinosis (NCL, Batten disease), *CLN8*, encodes for a protein regulating the lysosomal biogenesis; therefore, its deficiency leads to depletion of multiple lysosomal enzymes [7].

Historically LSD are classified according to the nature of the accumulating substrate such as mucopolysaccharidoses (MPS), sphingolipidoses, olygosaccharidoses (glycoproteinoses), glycogenoses, and lipidoses [6]. Although individually rare, combined, LSD have a frequency as high as 1 per 2200 live births [8]. While the majority of them are autosomal recessive, Fabry, Danon, and Hunter (MPS II) syndromes are X-linked [9]. In all known recessive LSD, ~50% reduction of the catabolic activity does not cause lysosomal storage phenotype in the cells; however, recent studies provided evidence that haploid insufficiency of several lysosomal enzymes is the major genetic cause of adult neurological diseases, primarily Parkinson’s, due to yet to be discovered pathological mechanisms.

About two-thirds of patients with LSD exhibit at least some neurological impairment [10] but the symptoms are heterogeneous and emerge at different ages. In children, these manifestations can include seizures, hearing loss, intellectual disability, neuromotor regression, developmental delay, and extra-pyramidal signs. In late-onset neurological LSD, adults can experience depression, dementia, psychosis, and other neurological deficits [9,11,12]. Neurological manifestations are typically present in MPS I, II, III, and VII, sphingolipidoses (G_M1_ and G_M2_ gangliosidosis, Gaucher disease, Niemann-Pick disease, metachromatic leukodystrophy, Krabbe disease and Farber disease), mucolipidoses, oligosaccharidoses (alpha- and beta-mannosidosis, fucosidosis, Schindler disease, aspartylglucosaminuria, sialidosis, and galactosialidosis), multiple sulfatase deficiency, and NCL [12].

Importantly, neurological LSD show genetic association and share multiple pathophysiological mechanisms with other neurodegenerative diseases, such as Parkinson’s disease (PD), Alzheimer’s disease (AD), dementia with Lewy bodies, and others. For instance, mutations in the gene *GBA*, encoding for β-glucocerebrosidase (Gcase), deficient in Gaucher disease (GD), are the highest genetic risk factors associated with PD [13,14,15]. Besides, pathogenic variants in *SCARB2*, encoding the receptor required for lysosomal targeting of GCase, and *PSAP*, encoding the precursor of saposin C, a requisite co-factor for enzymatic GCase activity on its natural substrate glucosylceramide, also enhance PD susceptibility [16]. The molecular basis of this association is still unclear but the increased levels of glucosylceramide have been linked to α-synuclein accumulation, the main cause of neuronal death in PD [17]. Other genes encoding for lysosomal enzymes and associated with PD susceptibility include *SMPD1* (acid sphingomyelinase), *CTSD* (cathepsin D), *ASAH1* (acid ceramidase), and GALC (lysosomal galactosylceramidase) [16,18]. 

On the other side, proteins that have been implemented into pathology of adult neurodegenerative diseases, such as tau protein and amyloid-β peptides, are also involved in the central nervous system (CNS) pathology in multiple LSD, including MPS (reviewed in [19]). Other examples include TAR-DNA binding protein 43 (TDP-43) and TMEM106B. TDP-43 that forms cytoplasmic aggregates in neurons of amyotrophic lateral sclerosis (ALS), AD, and frontotemporal lobar degeneration (FTLD) patients [20] also shows altered expression and mislocalization in the Niemann-Pick type C mouse and in a human neuronal model of the disease [21]. TMEM106B associated with frontotemporal dementia (FTD) and PD [22] is involved in lysosomal trafficking and function [23,24,25]. Finally, dominant mutations in *NAGLU* lead to a late dominant painful axonal sensory neuropathy and sensory ataxia, while recessive variants cause the lysosomal storage disease MPS IIIB [26]. 

Although some LSD can be treated with enzyme replacement therapy [27,28,29], substrate reduction therapy [30], pharmacological chaperones [31], or hematopoietic progenitor stem cell (HPSC) transplantation [32], the challenge in treating neurological LSD lies in unraveling an efficient therapeutic approach to cross the blood-brain barrier. Hence, studies that focus on understanding the neuropathophysiology of LSD are imperative to advance our understanding of the fundamental mechanisms of neuronal dysfunction in LSD and allow the development of novel therapeutic approaches to complement those treating the primary genetic defect.

## 2. Main Aspects of Central Nervous System (CNS) Pathology in Neurological LSD

Neurodegeneration and neuroinflammation manifesting with microgliosis and astrocytosis are described as the most common hallmarks of the brain pathology in neurological LSD with a propensity for an early onset [33,34,35,36,37,38,39]. In the mouse model of MPS IIIC, for example, astrocytosis and microgliosis are observed as early as at 4 months in the somatosensory cortex, when mice do not yet present behavioral abnormalities [35]. Heparan sulfate (HS)-derived oligosaccharides, the storage product common to all neurological forms of MPS, and presumably linked to CNS manifestations [40] is directly capable of triggering an inflammatory response in the CNS by acting on toll-like receptors (TLR) of microglial cells [41]. This results in release of pro-inflammatory cytokines such as TNF-α and MIP-1-α [35,42]. Moreover, HS fragments enhance integrin-based focal adhesions formation and activation in normal mouse astrocytes or in human neuronal progenitors, resulting in cell polarization and migration defects [43]. In the *Npc1^-/-^* mice, the mouse model of Niemann Pick type C 1 (NPC1), microglia contribute to the degeneration of Purkinje cells by engulfing and destroying their dendrites [44]. The same study also reported that microglia accumulated phagosomes and autofluorescent material that coincided with the degeneration of dendrites and Purkinje cells. In a healthy brain, microglial cells are crucial factors modulating neuronal and synaptic development, adult synaptic plasticity and regulation of neurogenesis [45]; however, in pathological conditions, their activation leads to the production of inflammatory cytokines that might lead to triggering of neuroinflammatory responses resulting in aggravated cell death. 

Similar to microgliosis, increased abundance and activation of astrocytes are observed in the majority of neurological LSD. Considering the important roles of astrocytes in regulation of synaptic strength and plasticity, astrocytosis could be an important contributing factor in the pathological CNS changes associated with these diseases. For example, in a mouse model of mucolipidosis IV (ML IV), increased glial cell derived neurotrophic factor (GFAP) immunoreactivity was observed at 2 and 3 months and coincided with early behavioural deficits. With disease progression, GFAP reactivity continued to increase until 7 months, when it could be responsible for alterations in synaptic plasticity in the absence of neuronal loss [36]. In the neuron-specific GCase knockout mouse model of Gaucher disease (*Gba*^f*lox/flox(Nestin-Cre)*^) astrogliosis as observed at P10 in layer V of cerebral cortex, lateral globus pallidus, and thalamic nuclei preceding the neuronal loss occurring at P14 [46]. Co-culturing wild-type primary mouse neurons with astrocytes and glia from a mouse model of CLN1 (*Ppt1^-/-^*), resulted in abnormal Ca^2+^ signaling, decreased neurite outgrowth and impaired neuronal survival [47]. However, culturing mutant neurons with healthy glia reversed the phenotype [48]. In a knock-in mouse model of CLN3 (*Cln3Deltaex7/8*), astrocytosis was more pronounced in the neocortex and thalamus than in other brain regions [49], while CLN6 mouse and oven models revealed progressive astrocytosis in several thalamic nuclei, somatosensory cortex, visual cortex, and caudate putamen [50,51]. Thus, it emerges, that at least in NCL, astrocytosis is a major pathological hallmark that precedes neurodegeneration.

In neurons affected by a LSD, the storage bodies are primarily confined to the perikarya, which might impair the transport of lysosomes, preventing them from merging with the early endosomes in the axon terminal [52]. The storage may also disrupt normal retrograde transport of vesicles carrying the synaptic proteins along the axon [52]. Furthermore, since lysosomes are the terminal compartments shared by the major autophagic and endocytic pathways of degradation [53], defective lysosomal homeostasis in LSD causes severe impairment of autophagy. Such instances have been reported in a variety of LSD including NCL, multiple sulfatase deficiency, MPS IIIA, MPS IIIC and NPC1 [35,54,55,56,57,58] leading to cell death [54], especially for neurons. Neuronal loss can be observed at different timepoints during the progression of the disease. In *Npc1^-/-^* mice, for instance, neuronal degeneration is widely distributed in the brain and can be observed as early as at 3 weeks in the lateral geniculate nuclei (LGNd) and ventral posterior medial (VPM) thalamic nuclei [38], and by 10 weeks of age, most Purkinje cells disappear [39]. In contrast, in MPS IIIC mouse model, NeuN-positive neurons in the somatosensory cortex are significantly reduced only at the age of 10 months [35].

Importantly, in multiple mouse models, signs of cognitive decline and behavioral abnormalities appear before the time when massive neuronal death emerges. For instance, MPS I mice at 8 months show severely affected motor function and cognitive decline but no signs of neuronal death, suggesting that the manifestations are caused mainly by a neuronal dysfunction [59]. In the MPS IIIC mouse, behavior abnormalities, such as hyperactivity, appear around 6 to 8 months of age and learning impairment at the age of 10 months, when neuronal loss is significant [35]. It is, thus, possible that at the initial stages of the disease, the neurological symptoms and behavioral deficits can be at least in part attributed to defects in synaptic transmission.

Indeed, one of such defects, synaptic pathology is a common feature for several animal models of LSD, including feline models of G_M1_ gangliosidosis [60,61], mouse model of NPC1 disease [38], the Twitcher mouse, the natural mouse model of Krabbe disease [62] and others. The described defects include scarcity of synaptic vesicles (SVs) in the axonal terminal leading to functional impairments in synaptic activity, impairment of the exocytosis [63], and endocytosis [62] of synaptic vesicles, loss of SNARE proteins, alterations in synaptic structures, dystrophic axons, and reduction or mislocalization of proteins involved in pre- and postsynaptic processes [34,57,64,65]. Below, these pathologies and their potential effects on brain function are described in detail. 

### 2.1. Impairment of Endo- and Exocytosis

Several studies have reported that endocytosis and exocytosis processes at the synaptic terminals are severely compromised by lysosomal storage. In the Twitcher mouse, neurons from the dorsal root ganglia, present a 2.5-fold decreased density of Ras-related protein Rab5-positive early endosomes. Besides, the percentage of Rab5+ vesicles containing the tyrosine kinase receptor A(TrkA) was decreased 1.4-fold [62]. Trk signaling is activated when a neurotrophin binds to the Trk receptor. The receptor further recruits the machinery responsible for its endocytosis and gets internalized in a lipid raft-dependent manner, forming a signaling endosome that traffics retrogradely to the cell body [66,67]. Retrograde transport of synaptophysin-positive vesicles was also decreased 1.5-fold, confirming that the early steps of endocytosis and retrograde transport of endocytic and synaptic vesicles are defective in the Twitcher neurons [62]. 

In hippocampal neuronal cultures of NPC1 mice labelled with the fluorescent dye FM4-64, the evoked vesicle exocytosis was impaired, with reduction in the fluorescence induced by potassium ion (61.9 ± 03% in *Npc1*^-/-^ mice; 71.6 ± 2.7% in wild- type (WT)). Components of the readily released pool (RRP) of synaptic vesicles were reduced at both excitatory and inhibitory *Npc1^-/-^* synapses [63]. The authors further tested synaptic vesicle turnover in *Npc1^-/-^* and WT neurons by delivering a set of 20 Hz stimuli followed by a test stimulus of 100 mV, 2 ms after various intervals, to allow for vesicle retrieval. In glutamatergic (excitatory) synapses, no difference between WT and NPC1 neurons was found, but the GABAergic (γ-aminobutyric acid, inhibitory) synapses in NPC1 cells exhibited impaired ability to retrieve synaptic vesicles after depletion [63]. In hippocampal neuronal cultures from MPS IIIA mice, fluorescent FM1-43 labelling experiments revealed that the dye uptake was reduced in the synaptic boutons of MPS IIIA neurons as compared to the WT, and that the exocytosis rate was significantly attenuated in MPS IIIA presynaptic terminals [57]. 

Together, these data provide strong evidence that impairment of endo- and exocytosis is a common trend shared among the LSD. It is known that lysosomal storage can affect vesicle trafficking and merging (such as merging of primary autophagosomes and lysosomes) by altering membrane lipid composition as well as interfering with a function of SNARE proteins [68,69,70]. It is thus plausible that similar mechanisms can also cause a compromised endo- and exocytosis of synaptic vesicles. Nonetheless, further experimentation is needed to establish a direct causality. 

### 2.2. Axonopathy

Dystrophic axons and demyelination have been reported in different neurological LSD. For instance, in a feline model of G_M1_ gangliosidosis, animals at the advanced stage of disease (7 to 9 months of age), exhibit slight distention of perikarya in the ventrolateral thalamocortical relay neurons as well as enlargements near the axon hillock area. The pyramidal neurons present scarcity of excitatory response that can be interpreted as a manifestation of abnormal integration of somatodendritic inputs [60]. Another study of this model, reported accumulation of complex lipids in membranous cytoplasmic bodies, causing the swelling of the neurons, which, however, had no impact on intracellular recordings or evoked excitatory and inhibitory postsynaptic potentials [61]. Axonal swelling was also observed in cultured hippocampal neurons from MPS IIIA mice at day in vitro 20–21 [57].

In NPC1 mice, large aggregates of presynaptic proteins, synaptophysin, synaptobrevin, VAMP2 and SNAP25, were observed in the subcortical grey and white matter (presumably in axonal enlargements) suggesting that a fraction of these proteins failed to reach the nerve terminals. Instead they accumulate within axonal spheroids in the white matter, providing strong evidence that axonal transport is disturbed in the *Npc1^-/-^* mice [38]. Signs of defective retrograde axonal transport, including unstable microtubules, and reduced dynein levels, were also detected in the dorsal root ganglia of the Twitcher mice [62]. Granular axonal spheroids and dystrophic axons have been described also in other LSD such as mannosidosis, G_M1_, and G_M2_ gangliosidosis, prosaposin deficiency, and NPC1 [38,61,71,72,73]. In NPC1, axonal spheroids were immunoreactive for GAD65/67 (a marker of GABAergic neurons) and synaptophysin in the VPM/VPL (ventral posterior nucleus/ventral posterior lateral) of the thalamic nuclei [38].

Demyelination of axons occurs in several neurological LSD, including Krabbe disease, metachromatic leukodystrophy (MLD), and ML IV [36,62,74]. In the Twitcher mouse, axonal demyelination in peripheral nerves is observed between postnatal day (P)15 to P30 [62,75,76]. In three autopsy cases of human MLD patients, demyelination manifested as a continuous diminution of myelin density, starting in late prelesional regions and following into early gliotic scar areas [74], whereas in ML IV (*Mcoln1^−/−^* mice), a thinning of axonal myelin sheaths coincided with malformation of the corpus callosum [36]. 

Thus, axonopathy appears to be a common trend among neurological LSD, especially sphingolipidoses. It is not known how the accumulation of specific substrates is linked to this phenomenon or whether other LSD also share the same characteristics. 

### 2.3. Changes in Synaptic Proteins

Multiple studies reported changes in the levels and/or distribution of synaptic proteins associated with lysosomal storage (Table 1). The majority of this research has been conducted using animal, primarily, mouse models, but recent advances in availability and improvements in quality of induced pluripotent stem cells (iPSC)-derived neuronal cultures made them a valuable alternative to investigate changes specific to human neurons. 

Several studies reported alterations in the levels and localization pattern of presynaptic vesicle membrane proteins with VAMP2 (vesicle-associated membrane protein 2) and synaptophysin, being among the most frequently studied. In the mouse models of MPS I, IIIA and IIIB, VAMP2 punctate staining was reduced and more diffused in the neurons of primary motor, somatosensory and parietal areas of the cerebral cortex as compared to their WT counterparts, while no difference was observed for synaptophysin [34]. The authors interpreted this as an indication for a possible rearrangement of presynaptic components without a loss in the overall number of synapses [34]. In contrast, in NPC1 mice, both VAMP2 and synaptophysin presented aggregated pattern and overall reduction in the ventral medial posterior nucleus of the thalamus and the ventral posterior lateral nucleus [38]. Another study in MPS IIIA mice, showed reduced levels of VAMP2 as well as of another presynaptic SNARE protein, SNAP25 [57]. They also demonstrated reduced levels of synaptic vesicles in the synaptic terminals accompanied by the presence of abnormal vacuoles and/or enlarged mitochondria [57]. Interestingly, the SNAP25 and VAMP2 mRNA levels were unchanged, suggesting that the decrease in both proteins in MPS IIIA neurons was rather caused by their increased degradation than impaired gene expression [57]. The authors hypothesised that increased degradation/reduced stability of SNAP25 and VAMP2 in MPS IIIA neurons was caused by a loss of α-synuclein and CSPα (cysteine string protein α), two abundant presynaptic proteins that act as chaperones and assist SNARE complex formation at synaptic terminals [77,78,79,80]. Interestingly, in a mouse model of the Finnish variant of CLN5 *(vLINCL(Fin)* early localized glial response at 4 months was followed by reduced expression of SNAP25, synaptophysin in thalamic nuclei such as VPM/lPL and LDNd while VAMP was found to be increased at 12 months. The authors propose a new sequence of events in the time course of neuronal loss, commencing in the cortex at 4 months, followed by subsequent neuronal loss in the thalamocortical system emerging at 12 months, which is opposite to what is observed in other animal models of NCL and propose synaptic pathology as the causality [81]. In another comparative study in early infantile NCL mouse model (*Ppt1^-/-^*) and a late onset CLN6^-/-^ mouse model, neuronal loss was observed in the thalamus early symptomatic stages followed by neuronal loss in cortical areas. Both mouse models displayed a concomitant reduction in the expression of synaptobrevin, synaptophysin and SNAP25 with VDAC and Pttg1 being the two most downregulated proteins. The authors propose VDAC and Pttg1 to be potential in vivo biomarkers for synaptic vulnerability in NCL [82]. On the other hand, a study by Hurtado et al., reported significantly reduced levels of synaptophysin in the thalamic nuclei in *Cln3^-/-^* mouse, while no reduction was observed in the cortical regions [83].

Reduced levels of synaptophysin were also reported in the mouse model of MPS IIIB [84], in human iPSC-derived neurons from MPS VII patients [85] and in the occipital and parietal lobes of sheep with NCL (CLN6) [86]. Two different mechanisms have been proposed to explain this reduction. Vitry et al. (2009) have shown that in the rostral cortex of MPS IIIB mice, synaptophysin was decreased as early as at 10 days after birth, preceding the onset of clinical symptoms [84], which prompted them to propose that, at the cellular level, the disease starts long before the appearance of the clinical symptoms. They suggested that in the neurons HS oligosaccharides activate the degradation of synaptophysin by the proteasome, which leads to its reduction [84]. In contrast, Bayo-Puxan et al. (2018) explained lower synaptophysin levels in MPSVII cultured neurons by the reduced gene expression [85]. In *Npc1^-/-^* mice, aggregations of synaptophysin were present in regions of the brain with reactive gliosis, including the striatum, substantia nigra, white matter tracts, and the thalamus [38]. The authors did not detect, however, a difference in the overall levels of protein expression, suggesting a redistribution rather than up- or down-regulation of these presynaptic markers [38]. In the CLN5 sheep model, neurodegeneration, and synaptic loss was more promiscuous in the motor cortex than in the cerebellum. In synaptosomes isolated from the motor cortex but not from cerebellum, several synaptic proteins, including α-synuclein, CSP-α, α-neurofascin, and ROCK 2 were decreased while calretinin and UBR-4 increased, suggesting the correlation of synaptic pathology with neuronal loss [87].

Homer-1, a protein enriched in the postsynaptic density of excitatory synapses was also significantly reduced in the MPS I, IIIA and IIIB mouse brains, suggesting that signalling at the postsynaptic density may also be altered [34]. Conversely, another study by Dwyer et al. (2017) showed that MPS IIIA mice presented enhanced puncta for postsynaptic density 95 (PSD-95) in the somatosensory cortex at P21. Increased PSD-95 was also confirmed by western blot of whole tissue homogenates and synaptosomes [64]. Furthermore, accumulation of heparan sulfate was found to increase the levels of AMPA (α-amino-3-hydroxy-5-methylisoxazole-4-propionic acid) receptor GluA2 on the cell surface, providing a possibility for arising of synaptic neurotransmission defects. 

Proteomic analysis of the hippocampus of MPS I mice have revealed reduced levels of many synaptic proteins, including syntaxin-1A, amphiphysin, complexin-1, synaptophysin, MAP1A, and MAP1B [59]. Syntaxin-1A is a part of the SNARE complex; amphiphysin and complexin-1 participate in the exocytosis of synaptic vesicles; synaptophysin is abundant in the synaptic vesicle membrane and is essential for their efficient endocytosis [88], MAP1A and MAP1B are microtubule-associated proteins involved in the filamentous cross-bridging between microtubules [89].

With an exception of Dwyer et al. (2017), who reported data showing induction of postsynaptic proteins PSD-95 and GluA2 in the neurons of MPS IIIA mice [64], and Baldo et al. (2015), who showed increased levels of PSD-95 in the hippocampus of MPS I mice [59], the majority of studies agree on a decrease in the levels of pre- and postsynaptic proteins potentially leading to synaptic function impairments in neurological forms of LSD. Further studies are however necessary, to both expand the number of disorders and models analysed and to clarify the mechanism underlying these changes. 

**Table 1:** Synaptic proteins altered in neurological lysosomal storage diseases.

LSD	Protein	Function	Change	Sample/Region	Reference
MPS I	Syntaxin-1A	SNARE complex	Reduction	Hippocampus	Baldo et al. (2015) [59]
	Amphiphysin	Exocytosis of synaptic vesicles	Reduction	Hippocampus	
	Complexin-1	Exocytosis of synaptic vesicles	Reduction	Hippocampus	
	Synaptophysin	Synaptic vesicle membrane protein involved in endocytosis	Reduction	Hippocampus	
	MAP1A	Microtubule cross-linking protein	Reduction	Hippocampus	
	MAP1B	Microtubule cross-linking protein	Reduction	Hippocampus	
	PSD-95	Postsynaptic density protein	Increased	Hippocampus	
	VAMP2	Vesicle-associated membrane protein 2 in the synaptic vesicles	Reduction	Primary motor, somatosensory and parietal areas of cerebral cortex	Wilkinson et al. (2012) [34]
	Homer-1	Protein in the postsynaptic density of excitatory synapses	Reduction	Primary motor, somatosensory and parietal areas of cerebral cortex	Wilkinson et al. (2012) [34]
MPS IIIA	SNAP25	t-SNARE	Reduction	Synaptosomes	Sambri et al. (2017) [57]
	VAMP2	Vesicle-associated membrane protein 2 in the synaptic vesicles	Reduction	Primary motor, somatosensory and parietal areas of cerebral cortex; synaptosomes	Wilkinson et al. (2012) [34]; Sambri et al. (2017) [57]
	Homer-1	Protein in the postsynaptic density of excitatory synapses	Reduction	Primary motor, somatosensory and parietal areas of cerebral cortex	Wilkinson et al. (2012) [34]
	PSD-95	Postsynaptic density protein	Increased	Cortical layers I, II/III and V	Dwyer et al. (2017) [64]
	α-Synuclein	Presynaptic chaperone	Decreased	Cultured neurons and brain homogenates	Sambri et al. (2017) [57]
	CSPα	Presynaptic chaperone	Decreased	Cultured neurons and brain homogenates	
MPS IIIB	Synaptophysin	Synaptic vesicle membrane protein involved in endocytosis	Reduction	Rostral cortex	Vitry et al. (2009) [84]
	VAMP2	Vesicle-associated membrane protein 2 in the synaptic vesicles	Reduction	Suprachiasmatic Nucleus; Primary motor, somatosensory and parietal areas of cerebral cortex	Canals et al (2010) [65]; Wilkinson et al. (2012) [34]
	Homer-1	Protein in the postsynaptic density of excitatory synapses	Reduction	Primary motor, somatosensory and parietal areas of cerebral cortex	Wilkinson et al. (2012) [34]
MPS VII	Synaptophysin	Synaptic vesicle membrane protein involved in endocytosis	Reduction	iPSC-derived neurospheroids	Bayo-Puxan et al. (2018) [85]
	GAD67	Enzyme that catalyzes the production of GABA	Reduction	iPSC-derived neurospheroids	
Niemann-Pick Type C	Synaptophysin	Synaptic vesicle membrane protein involved in endocytosis	Aggregation	Striatum, substantia nigra, white matter tracts and thalamus	Pressey et al. (2012) [38]
	VAMP2	Vesicle-associated membrane protein 2 in the synaptic vesicles	Aggregation	Striatum, substantia nigra, white matter tracts and thalamus	
Krabbe	Dynein	Retrograde transport of synaptic vesicles	Reduction	Dorsal Root Ganglia Neurons	Teixeira at al. (2014) [62]
Gaucher	α-synuclein	regulation of synaptic vesicle trafficking and neurotransmitter release	Accumulation	Striatum	Ginns et al. (2013) [37]
CLN3	Synaptophysin	Synaptic vesicle membrane protein involved in endocytosis	Reduction	Thalamic nuclei	Hurtado et al., (2017) [83]
CLN5	α-synuclein	regulation of synaptic vesicle trafficking and neurotransmitter release	Reduction	Synaptosomes	Amorim et al., (2015) [87]
	CSP-α	Presynaptic chaperone	Reduction	Synaptosomes	
	α-neurofascin	Cell adhesion molecule	Reduction	Synaptosomes	
CLN6	Synaptophysin	Synaptic vesicle membrane protein involved in endocytosis	Reduction	Occipital and parietal lobes	Kanninen et al. (2013) [86]
	Syntaxin-6	Intracellular vesicle trafficking	Reduction	Occipital lobe	

LSD, lysosomal storage diseases; MPS, mucopolysaccharidoses; PSD-95, postsynaptic density 95; CSP-α, cysteine string protein α; CLN, neuronal ceroid lipofuscinosis.

### 2.4. Alterations in Generation and Recycling of Synaptic Vesicles

Synaptic Vesicles (SVs) store neurotransmitters such as acetylcholine, glutamate, glycine, and GABA that are released at the synaptic terminal upon an action potential that causes Ca^2+^ influx and results in their exocytosis into the synaptic cleft [90]. SVs and their precursors are transported via microtubules [91] either by the motor protein kinesin (anterograde transport from the soma to the periphery) [92], or by dynein (retrograde transport from the periphery to the soma) [93]. At the nerve terminal, SVs can be found in three distinct pools: the reserve pool, the recycling pool, and the RRP [94]. As described in the sections above, lysosomal storage influences the homeostasis of the protein machinery involved in the synaptic vesicle trafficking and recycling, and affects vesicles in all three pools. For instance, impairment of the early steps of endocytosis and the retrograde transport of endocytic and synaptic vesicles were reported for the dorsal root ganglia neurons of a Twitcher mouse [62]. The authors linked vesicle trafficking deficits with decreased levels of dynein and reduced microtubule stability. Reduction in the number of SVs in both excitatory and inhibitory synapses was also described in NPC1 neurons, mostly affecting the RRP [63]. The same study also reported evoked vesicle exocytosis suggestive of functional presynaptic defects [63]. Interestingly, the degree of impairment of synaptic vesicle turnover was different in excitatory and inhibitory neurons. At glutamatergic synapses, the synaptic vesicle turnover was either normal or impaired just for a short period of time (<0.2 s), while at GABAergic synapses, the consistent blockage of the turnover was observed [63]. In a similar fashion, neurons from MPS IIIA mice presented reduced density of synaptic vesicles at the synaptic terminals associated with a severe functional impairment in the synaptic activity [57]. Docked SVs were reduced by 30% in cortical neuronal cultures from the CLN1 mice [95].

Based on these studies, it seems that there is a reduction in number of synaptic vesicles at the synaptic terminals of LSD neurons caused by impaired vesicle turnover and intracellular trafficking that lead to functional impairment in synaptic transmission.

### 2.5. Defects in Synaptic Spines

Synaptic spines are small protrusions on the plasma membrane of the dendritic branches responsible for receiving ~95% of excitatory inputs in pyramidal neurons [96,97,98]. Typical excitatory spines have a small head (~1 µm in diameter, <1 fL volume) and are separated from the dendrite by a thin (<0.2 µm in diameter) neck [97,99]. Dendritic spines are important for synaptic plasticity and are believed to be the preferential site for the induction of long-term potentiation [100,101]. Therefore, alterations affecting their shape or number can directly reflect on the cognitive defects observed in LSD patients. The lysosomes play an important role in the process of synaptogenesis, potentiation and pruning of the spines. Backpropagating action potentials elicit Ca^2+^ release from lysosomes to dendrites and trigger the fusion of the lysosome to the plasma membrane, resulting in the release of Cathepsin B. Cathepsin B then increases the activity of matrix metalloproteinase 9 (MMP-9), an enzyme involved in extracellular matrix (ECM) remodeling and synaptic plasticity. Inhibiting either lysosomal Ca^2+^ signaling or Cathepsin B release prevents dendritic spine growth [102], whereas blocking Ca^2+^-dependent fusion of lysosomes to the plasma membrane leads to an overall decrease in spine number and increase of their length resulting in filopodia-like spines [103]. 

Since lysosomal homeostasis plays an important role in maintaining the synaptic spines, and considering that impaired synaptic activity is observed in animal models of neurological LSD, one can expect that studies involving these models or post-mortem human samples should include analysis of synaptic spines. However, only a few reports took this into consideration. In 1976, using Golgi staining of neurons from either biopsies or post-mortem brain samples from G_M2_ gangliosidosis patients, Purpura & Suzuki revealed that dendritic spines on pyramidal and non-pyramidal neurons were abundant and normal in overt appearance [104]. However, a loss of spines was detected in samples of other patients with G_M2_ gangliosidosis and NCL [104]. Loss of spines in NCL (PPT1 deficient mice) was also documented by Bible et al. [105]. Using Golgi silver-impregnation of layer II/III pyramidal neurons in the primary somatosensory cortex of MPS IIIA mouse at P21, it was demonstrated that synaptic spine density was not affected, but there was an increase in the width of the spine heads (mushroom spines) [64].

Two studies in NPC1 neurons obtained controversial results: while Xu et al. (2010) reported no differences in spine density [63], Tiscione et al. (2019) observed that NPC1 deficiency results in a decrease in neuronal spine density [106]. Thus, based on the published data, it appears that alterations of synaptic spines do not follow the same trend among all neurological LSD.

### 2.6. Changes in Postsynaptic Density

The postsynaptic density (PSD) is the most prominent excitatory postsynaptic component situated at the distal tip of the spine head [107]. It is identified as an electron-dense structure extending 35–50 nm into the cytoplasm beneath the plasma membrane [108,109]. The surface area of the PSD correlates with spine head volume, the total number of presynaptic vesicles [110], and the number of vesicles docked at the active zone [111,112,113]. The structure and composition of PSDs change during the maturation of synapses and expression of major PSD proteins, such as PSD-95, calcium/calmodulin-dependent protein kinase type II subunit alpha (CaMKIIα), and AMPA receptor subunits are increased during neuronal development [114,115]. Alterations in the levels of PSD components are known to cause neurological and psychiatric disorders [116] and, therefore, might be common to LSD and related neurodegenerative diseases. 

The reduction in size of PSDs has been described in 3 mouse models of Gaucher disease (knock-in mice homozygous for human L444P and R463C missense mutations, and a conduritol-β-epoxide (CBE)-induced model) [37]. Elongated PSDs in asymmetric excitatory synapses were described in the mouse model of ML IV [36] while no changes in the postsynaptic components were observed in NPC1 [38,63]. In general, spines with larger heads also have bigger PSDs, indicating stronger synapses and the promotion of long-term potentiation [116]. On the other hand, immature spines (filopodia) with smaller, or even absent PSDs, are a sign of weak synapses and could account for cognitive deficits observed in many types of neurological LSD. 

## 3. Functional Synaptic Defects

Alterations in synaptic neurotransmission in LSD have recently received more attention and have been proposed to play a major role in pathophysiology of these disorders. Most studies could detect the emergence of defects in synaptic neurotransmission before the commencement of neurodegeneration and neurobehavioral impairments. For example, in 21-day-old MPSIIIA mice, electrophysiological recordings of miniature excitatory postsynaptic currents (mEPSCs) from layer II-III of the somatosensory cortex revealed fewer larger events and a smaller average mEPSC amplitude. However, the mEPSC frequency remained unaltered in comparison with control animals consistent with similar numbers of dendritic spines. In the same animals, field hippocampal synaptic neurotransmission from CA1 cells remained unaltered in response to increasing stimulation of Schaffer collateral (SC), suggesting a reduced synaptic strength possibly at the postsynaptic site [64]. Interestingly, in the same model, field recordings in hippocampal slices from 6-month-old animals revealed reduced synaptic activity. A decrease in the fEPSP (field excitatory postsynaptic potential) slope with increasing stimulus intensity and presynaptic response amplitude was detected suggesting a presynaptic deficit, consistent with the reduction of synapse density and a number of synaptic vesicles per synapse at the presynaptic terminals as detected by electron microscopy (23). In cultured rat hippocampal neurons, lysosomal trafficking was found to be a crucial modulator of postsynaptic spine remodeling [117]. Inhibition of lysosomal proteolysis by cysteine protease inhibitor leupeptin resulted in the reduction of mEPSC frequency, but not of the amplitude. A concomitant reduction in the number of spines at excitatory synapses was also observed, suggesting that local lysosome-dependent degradation of synaptic proteins is essential for synaptic activity-mediated remodeling of synaptic spines [117]. Early synaptic impairments have been also reported in a mouse model of CLN1 (*Ppt1^-/-^*), in which the developmental switch in N-methyl-D-aspartate(NMDA) receptors from GluN2B to GluN2A was found to be arrested [118]. Furthermore, SAP-102 and PSD95 proteins interacting with GluN2B and GluN2A, respectively, were significantly reduced at P33-P60 and alterations in the kinetics of the NMDA excitatory postsynaptic currents (EPSCs) indicated longer activation times of GluN2B receptor [118].

Mechanisms by which lysosomal storage affects basal synaptic transmission and synaptic plasticity are not completely understood; however, a substantial body of research provides evidence that these changes lead to neurodegeneration and neurobehavioral symptoms in the affected patients and animal models. One example of such impact has been provided by a study involving a mouse model of NPC1 [119]. NPC1 mice display steeper hippocampal fEPSP slope in response to amplified SC-CA1 stimulation intensities, indicating increased basal synaptic neurotransmission. Additionally, paired pulse facilitation was found to be increased in NPC1 mice, also indicating the possibility of an increased presynaptic release. The authors concluded that an increase in basal synaptic transmission could lead to chronic neuronal excitotoxicity and frequent seizures in NPC1 patients [119]. In the same animals, hippocampal long-term potentiation (LTP) has been significantly reduced, which could potentially explain the cognitive impairments observed in NPC1 mice [120] and patients [121]. Overall, a decrease in LTP, in combination with induced basal transmission, could lead to a distinct pattern of neurobehavioral symptoms in the NPC1 patients. 

Both human NPC1 patients and mice show drastic degeneration of cerebellar Purkinje cells [122]. Interestingly, Sun et al. (2011) reported that this coincides with an increase in parallel fiber-Purkinje cell basal synaptic transmission, and a concomitant reduction in long-term depression (LTD), leading to increased vulnerability of these particular cell types [123]. The authors propose that these synaptic defects stem from the decrease in ATP/adenosine release and deactivation of A1 receptors [123]. However, a study by Xu et al. (2010) reports that defects in synaptic vesicle turnover in NPC1 mice were more severe in inhibitory than in excitatory synapses. Similarly, a reduction in the amplitude of evoked inhibitory postsynaptic currents (IPSCs) with increasing stimulation indicated decreased synaptic depression at inhibitory synapses, while no changes in synaptic depression were found at excitatory synapses [63]. Further evidence for vulnerability of GABAergic synapses in LSD comes from a recent study in which cathepsin D (CTSD), an aspartate protease whose genetic deficiency is associated with a subtype of NCL10 was found to be differentially regulating synaptic vesicle recycling processes in GABAergic and glutamatergic synapses [124]. Acute pharmacological inhibition of CTSD led to a decrease of the RRP size in GABAergic but not glutamatergic synapses, causing the decrease in the amplitude of the IPSCs, but not the EPSCs. The authors proposed that CTSD is therefore an important regulator of synaptic vesicle recycling, and that its deficiency blocks this process causing neuronal dysfunction and the epilepsy prone behavior in NCL10. In another juvenile mouse model of NCL (*Cln3^Δex1-6^*), the authors reported specific synaptic neurotransmission deficits in multiple brain areas correlating with anxiety and memory impairments [125]. Whole cell patch clamp recordings from principal neurons (PNs) in the basolateral amygdala (BLA) displayed reduced frequency of mIPSCs (miniature inhibitory postsynaptic currents) and sIPSCs (spontaneous inhibitory postsynaptic currents) as well as reduction of peak amplitude in evoked GABAergic IPSCs. These observations indicated a higher vulnerability of the inhibitory interneurons at least in the BLA that could in part account for the observed increased anxiety. The authors also reported reduction in the frequency and amplitude of mIPSCs and sIPSCs in the dentate gyrus neurons of the hippocampus, suggesting compromised presynaptic release. The authors propose that the two affected brain regions mediate different behavioural impairments with a common signature of a GABAergic deficit [125]. At the network level, a significant decrease in short wave ripples in the hippocampus (a characteristic oscillation often implicated in memory consolidation) over the course of the disease was found in a CLN3 mouse model (*Cln3^-/-^*) suggesting that neuronal network defects in conjunction with synaptic deficits aggravate with age [126].

Enzyme replacement therapy (ERT) remains the most accepted treatment regimen for LSD although it is widely accepted that systemic ERT cannot effectively manage neurological indications due to inability of the recombinant enzyme to pass the blood-brain barrier. Interestingly, a study by Stroobants et al. [127] reports that systemic administration of recombinant human α-mannosidase through tail vain injections for 7 months (starting at 2 and continuing until 9 months) in an immune-tolerant mouse model for α-mannosidosis improved spatial cognitive performance in the Morris water maze task. LTP induced by stimulation of SC to CA1 neurons in the hippocampus was also found to be increased with a concomitant reduction in primary substrate storage and neuroinflammation. The authors propose that long-term ERT might thus represent a potential treatment option for neurological manifestations of LSD. However, the precise mechanism of action of α-mannosidase and identification of its substrates in the CNS requires further elucidation. It is tempting to speculate that the delivered recombinant enzyme possibly catabolizes the accumulated substrate at peripheral sites, reducing neuroinflammatory responses in the CNS. 

## 4. iPSCs as An Emerging Model to Study Human Neuronal Dysfunction in LSD

While the majority of animal models for LSD closely recapitulate disease phenotypes, they are limited in encompassing a range of mutations specific for human patients. Additionally, loss of function or toxic gain of function models might present phenotypes in the animal models that are actually not observed in human patients. For example, murine models of MPS IIIB and MPS IIIC develop urinary retention, which is atypical of human patients [35,128]. Patient-derived primary fibroblasts carry naturally occurring mutations within a specific genetic background that allows to study storage phenotype, as well as residual activity, trafficking, processing, stability, and other properties of the mutant lysosomal enzymes [129]. However, degeneration and other prominent phenotypes typically observed in neurons would not be closely recapitulated because of the inherent metabolic differences between the two cell types. To this end, neurons derived from virally reengineered fibroblasts to create iPSCs emerge as an attractive model for studying patient-specific neuronal dysfunction mechanisms in lysosomal storage disorders. The iPSC models of LSD are also robust cellular tools for mining valuable information on disease mechanisms and therapeutic targets.

The wide variety of methods adopted to achieve differentiation of iPSCs to a panoply of different neuronal types is another major advantage, as it allows to study mechanisms of dysfunction in the particular cell types, and detect the one most vulnerable during the disease progression. Examples of such “Achilles” heel include midbrain dopaminergic neurons in Gaucher disease [130], Purkinje cells in NPC1 and α-mannosidosis [131,132], medium spiny GABAergic neurons in G_M1_ gangliosidosis [133], hippocampal neurons (from the dentate gyrus and CA3) in MPS and NCL, and cortical pyramidal neurons in NCL, Tay-Sachs and MPS [65,85,134,135]. Recent advances in the above-mentioned diseases pertaining to the use of iPSC models are described in detail below.

Neurological Gaucher disease—while type 2 (acute neuronopathic) and type 3 (chronic neuronopathic forms) of Gaucher disease have been primarily associated with neurological manifestations, recently, mutations in β-glucocerebrosidase (Gcase) encoding gene *GBA-1*, have been associated in heterozygosity with a high recurrence of Parkinson’s disease (PD). *GBA-1* haploinsufficiency has been proposed to generate partial lysosomal dysfunction leading to accelerated protein aggregation and neuronal death in midbrain dopamine rich areas. The iPSC-derived dopaminergic (DA) neurons from idiopathic PD patients were generated to test this hypothesis [136]. These iPSC DA neurons were found to recapitulate predicted GD hallmarks, such as lysosomal dysfunction, Gcase deficiency, glucosylceramide storage, and α-synuclein (α-syn) accumulation as seen in PD patients. Additionally, a non-inhibitory small molecule moderator of Gcase-”758” could increase Gcase activity and reduce amyloidogenic accumulation of α-syn in the body of tyrosine hydroxylase-positive DA neurons [136]. The iPSC-derived neurons from GD patients have shown several electrophysiological abnormalities, including a reduced action potential (AP) firing in response to depolarizing current stimulations, a lesser proportion of GD neurons firing APs, a significantly less negative resting membrane potential and reduced AP amplitude [137]. The authors further showed that treatment of control iPSC neurons with the Gcase inhibitor CBE results in similar phenotype, implicating that Gcase is behind the abnormal electrophysiological properties of the neurons [137]. Another study involving iPSC-derived neurons from a neuronopathic type II GD patients demonstrated a reduced stability and expression of the transcription factor EB (TFEB), the master modulator of lysosomal biogenesis and autophagy. Additionally, the study recapitulated typical hallmarks of GD neurons, such as autophagy block, defective autophagic clearance, lysosomal depletion, and decreased rapamycin toxicity threshold [138]. In a following study by the same group, the authors proposed a mechanism of the autophagy-lysosomal mediated dysfunction in the GD neurons. The authors report an increased activity of mTORC1 (mammalian target of rapamycin complex 1) concomitant with a reduced expression of TFEB, and increased phosphorylation of its downstream targets 4EBP1 and RPS6. Interestingly, the authors found that inhibiting mTORC1 by Torin1 upregulated lysosomal expression and improved autophagic clearance, suggesting that mTORC1 can be a potential therapeutic target for treating neurodegeneration in CD patients [139]. New insights continue to emerge with a generation of novel GD iPSC cell lines [140,141,142], enabling researchers to advance understanding of the neuronal signatures of this disease. 

A recent study has tested whether WT mouse iPSC-derived neuronal progenitor cells (NPCs) can be used for treatment of the disease in the mouse GD model [143]. The authors used the fraction of NPCs expressing Very Late Antigen 4 (VLA4, integrin α4β1), a factor that permits entry of NPCs in the brain after intravenous infusion. After engraftment of the infused VLA4+ NPCs, the disease progression in the mice, including weight loss and hindlimb paralysis, was delayed. The procedure has also increased the life span and reduced neuroinflammation as measured by reduced expression of CD68, GFAP (glial fibrillary acidic protein), and TNF-α in the brain tissues. Additionally, the induced expression of neurotrophic factors such as brain derived neurotrophic factor (BDNF), glial cell derived neurotrophic factor (GDNF), and neurotrophic factor 3 (NT3) were found to be significantly increased, laying the basis for a considerably reduced neurodegeneration [143]. Whether a similar engraftment of normal or autologous gene corrected iPSC-derived NPCs could also alleviate disease phenotypes in GD patients warrants further investigation. 

Niemann-pick type C—neurological impairments of early onset NPC1 include cerebellar ataxia, strongly implicating the loss of Purkinje cells in the cerebellum [144]. Although, there have been no published reports yet exclusively on iPSC derived Purkinje neurons, a study by Lee et al. [145], reported that iPSCs-derived neurons from NPC1 patients accumulated cholesterol and sphingosine, and had reduced levels of sphingosine kinase (SphK) activity and VEGF (vesicular endothelial growth factor) levels. The authors proposed that sphingosine accumulation was caused by inactivation of the VEGF/SphK activity, leading to inhibition of autophagosome-lysosome fusion. SphK activity could also be rescued by co-culturing NPC1 iPSC-derived neurons with VEGF*^tg^* bone marrow mesenchymal stem cells, or by treating them with recombinant VEGF, with a concomitant reduction in sphingosine accumulation, autophagosome accumulation, an increase in calcium release, and improvement of cell survival. 

In another study, electrophysiological analyses of iPSC-derived neurons from NPC1 patients revealed that peak current densities of Na^+^, K^+^, and Ca^2 +^ currents were similar to those of control cells [146]. However, a significantly less negative resting membrane potential and a decrease in the Ca^2+^ influx mediated by AMPA receptors were observed in the NPC1 neurons. The GluA1 and GluA2 subunits of AMPA receptors were also found to be upregulated in the NPC1 cells. Since fast synaptic transmission at the synapses is dependent on clustering and positioning of AMPA receptors, it is possible that increased cholesterol levels disrupt the organization of lipid rafts and contained ion channels at the synapse, thereby affecting internalization of GluA2 receptors. Alternatively, reduced Ca^2+^ influx in NPC1 could originate from an impairment in the trafficking of the AMPA receptors to the synapses or cause an imbalance in the uncoupling of the GluA2 subunits. Nevertheless, these studies highlight the therapeutic potential of ion channel modulators that could possibly ameliorate synaptic neurotransmission in NPC1 neurons. 

Neuronal ceroid lipofuscinosis—similar to the brain neurons, neuronal cells derived from a CLN5 patient’s iPSCs displayed accumulation of autofluorescent storage material and aggregates of the subunit C of the mitochondrial enzyme ATP synthase [147]. Sizes of the ER compartment and lysosomes, but not the lysosomal numbers, significantly increased as compared with those of healthy iPSC derived neurons. Additionally, BODYPI-LacCer labeling experiments demonstrated alterations in the neuronal sphingolipid transport, highlighting the utility of this cell-based model for studying mechanisms of neuronal dysfunction in NCL. The more recent study reported a reduced size of cerebral organoids derived from CLN10 patient’s iPSCs with concomitantly decreased mRNA levels of FOXG1 (forebrain marker), SATB2 (later born superficial layer identity neuronal marker), and TBR1 (early born deep layer identity neuronal marker) [148]. Mutant cerebral organoids also displayed reduced levels of CTSD and increased astrocytosis. However, no subsequent increases in the number of apoptotic cells or RIP1 and RIP3 (kinase receptor interacting proteins 1 and 3) were found. Proteomic analysis revealed the reduction in the expression of GABA receptor 2 (GABRA2) and dopamine receptor1 (DRD1), suggesting that they could be potential therapeutic targets [148]

G_M1_ gangliosidosis—the diverse etiology associated with G_M1_ gangliosidosis prompts the use of specific subtype of iPSC-derived neurons, such as cortical neurons (for severe early onset Types 1 and 2 of the disease) or medium spiny neurons (for the milder type 3) to closely recapitulate in vivo cell pathology. Nonetheless, iPSCs-derived NPCs (generalized neurons and glial cells) of G_M1_ gangliosidosis patients were found to mimic expected disease phenotypes, such as deficient β-galactosidase activity and G_M1_ accumulation concomitant, with an increased number and size of lysosomes and activation of inflammasomes [149]. Nevertheless, much remains to be understood regarding pathology in specific neuron subtypes, as well as in glia and other non-neuronal brain cells. Since G_M1_ ganglioside accumulation has also been associated with other age-related proteinopathies, e.g., Alzheimer’s disease [150,151], studying whether G_M1_ gangliosidosis iPSC-derived neurons reveal other storage material, such as α-syn, tau, or amyloid-β protein aggregates could help illuminate pathological pathways common for these disorders.

Mucopolysaccharidoses—the heterogeneity in the severity of neurological impairments and the multitude of implicated lysosomal enzymes, makes it one of the most complex disease to recapitulate in iPSC cell models. Neural stem cells (NSCs) derived from MPS I patient iPSCs closely follow predicted features of the disease phenotype, such as decrease in IDUA activity and increased accumulation of heparan and dermatan sulfate, presence of enlarged lysosomes, and differential expression of extracellular matrix genes revealed by transcriptome analysis [134]. However, whether differentiated neurons from these NSCs would mimic synaptic hallmarks associated with MPS I, such as decreased PSD-95 expression, remains to be studied [59].

MPS II, or Hunter syndrome, caused by deficiency of α-L-iduronate sulfatase (IDS), shares common spectrum of accumulated glycosaminoglycans (GAGs) and clinical symptoms with MPS I (except the absence of corneal clouding in the MPS II patients). Importantly, in both diseases, mildly affected patients do not show CNS abnormalities. The iPSCs generated from MPS II patients’ peripheral white blood cells were differentiated into neurons, oligodendrocytes, and astrocytes [152] that all showed structural abnormalities resembling lysosomal storage phenotype and GAG accumulation. Treating the cells with recombinant IDS (Elaprase^R^) used for ERT in MPS II, patients failed to decrease GAG accumulation, highlighting importance of iPSC-derived cells as a tool for developing better therapeutic molecules for neurons and glia [152].

MPS III, or Sanfilippo Syndrome, has very little somatic implications, but causes early-onset CNS degeneration across all the 4 subtypes (MPS IIIA to MPS IIID). In a study by Canals et al. [65], MPSIIIC iPSCs-derived neurons displayed the predicted disease phenotypes, e.g., GAG accumulation, reduced HGSNAT activity, and cytoplasmic vacuoles. Interestingly, recording of spontaneous activity, as measured by calcium fluorescence imaging, revealed a reduced number of repeating firing episodes of large amplitude. Upon further evaluation, the average number of firing episodes within the 30-min recording period was significantly reduced, with a concomitant decrease of the fraction of neurons that exhibited at least one spontaneous burst. Using a network activity modeling approach to create a functional map of neuronal interactions, the number of connections made by MPS IIIC iPSCs was found to be significantly weaker with increasing time in culture, in comparison to iPSCs-derived neurons from healthy controls. The reduction in network activity was consistent with the reported reduction in spontaneous miniature EPSCs found in the somatosensory cortex of P22-P23 MPS IIIA mice and reduction in neuronal activity in hippocampal slices of 6-month-old MPS IIIA mice [57,64].

Since, MPS III disorders involve neurobehavioral changes, strong cognitive decline, and memory impairment, most studies in mouse models concentrate on pathology of the somatosensory cortex, amygdala, and the hippocampus. Thus, it would be interesting to characterize spontaneous single cell activity in MPS III iPSC-derived cortical and hippocampal neurons, to delineate any observable differences or assign a common neuronal signature. These neuronal signatures can then be harnessed as biomarkers and enable screening of compounds with therapeutic relevance.

To enable identity of neuronal pathological phenotypes associated with MPS VII, Puxan et al. [85] generated 3-dimentional neurospheres from two patient iPSCs cell lines. The MPS VII iPSC-derived neurons displayed the typical features associated with the disease phenotype like deficient β-glucuronidase activity, increased GAG accumulation, ultrastructural aberrations in the lysosome, expanded endocytic compartments, and increased autophagosomes [85]. Ratiometric calcium imaging demonstrated that MPS VII iPSC neurospheroids displayed levels of cytosolic calcium and KCl-induced depolarization similar to those of normal controls, suggesting identical synaptic vesicle trafficking process. However, upon measuring spontaneous activity, a lesser number of MPS VIII neurospheroids were found to elicit calcium signals with a significantly reduced peak amplitude. A weaker neuronal connectivity pattern was also detected in MPS VII neurospheroids, indicating the possibility of an inherent altered synaptic activity [85]. 

Overall, iPSC models of LSD are generating a wealth of crucial information, especially owing to their amenability to harbor disease-causing mutations in a genetic background that is relevant for the disease. Additionally, the convergence of several mechanistic pathways common to both lysosomal storage and adult neurodegenerative disorders has led to the emergence of iPSC-based models as robust tools for delineating fundamental mechanisms of neuronal dysfunction common to disease spectra, allowing identification of valuable synaptic targets.

## 5. Conclusions

Apart from the classical hallmarks of neurological forms of LSD, such as neuroinflammation, impaired autophagy, disruption of lysosomal homeostasis, and neuronal death, much about the neuronal pathology remains to be explored. This mainly implies the neuronal functional defects that could be causing the behavioral and neurological impairments in LSD patients in the early stages of disease progression.

**Figure 1:**
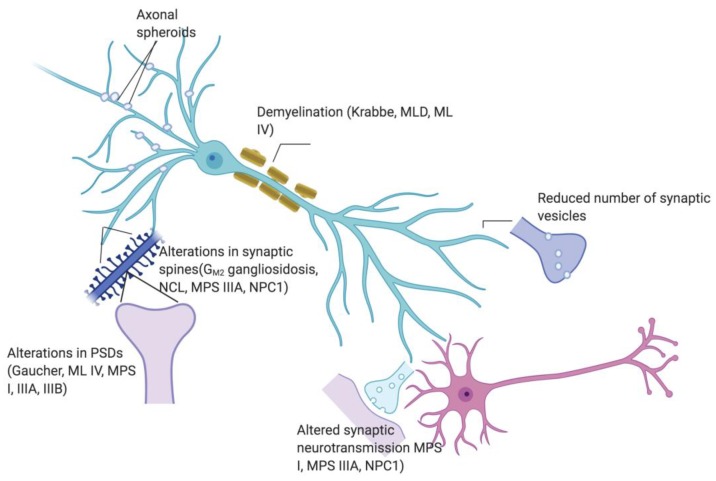
Scheme showing synaptic abnormalities (presence of axonal spheroids, demyelination, reduced synaptic vesicles, altered synaptic neurotransmission, altered spine morphology and alterations in post synaptic densities) in respective relevant LSD.

Synaptic phenotypes share some similarity across different types of LSD (Figure 1). For instance, synaptophysin appeared to be reduced in MPS I, MPS IIIB, MPS VII, and CLN6 and VAMP2 in MPS I, MPS IIIA and MPS IIIB, while both proteins are aggregated but not reduced in NPC1. Since the majority of presynaptic proteins associated with synaptic vesicles, as well as endocytosis and exocytosis machinery are reduced, it is tempting to hypothesize that impaired synaptic transmission stems from impaired synaptic vesicle trafficking. On the other hand, the growth and dynamics of synaptic spines seem to possess diverse features in different LSD.

Published data provide evidence that synaptic architecture abnormalities lead to functional defects affecting different subsets of neurons and, consequently, altering their electrophysiological activity; however, a considerable gap exists in available relevant information on progression of synaptic neurotransmission impairments at each stage of the disease. For instance, it is unclear whether animal models of LSD possess inherent synaptic deficits or synaptic neurotransmission progressively deteriorates with age. There is also no robust study delineating whether synaptic deficits precede and lead to neuronal death. Thus, there is a pressing need to identify crucial time windows for development of neurotransmission deficits that may help generate important insights into targeting therapies at the synaptic level and identifying synaptic modulators with therapeutic potential. 

The emergence of iPSCs has led to a revolution in approaching human mutation-specific phenotypes of LSD. However, most studies aim at recapitulating already established disease specific hallmarks that have been documented in animal models of LSD. While, the former aspect is imperative to justify the quality and fidelity of the model, studies using iPSC-based models to assign patient-specific neuronal signatures are still mainly missing. These neuronal signatures can then be used as biomarkers to enable medium-throughput screening strategies for therapeutics that act specifically on CNS indications of LSD. The iPSC-based models might therefore emerge as the first step in revolutionizing personalized medicine in the field of LSD.
